# Accessibility, neighborhood socioeconomic disadvantage and expenditures on electronic gambling machines: a spatial analysis based on player account data

**DOI:** 10.1186/s12942-024-00379-2

**Published:** 2024-08-31

**Authors:** Jani Selin, Pasi Okkonen, Susanna Raisamo

**Affiliations:** Finnish Institute for Health and Welfare, P. O. Box 30, 00271 Helsinki, Finland

**Keywords:** Electronic gambling machines, Accessibility, Expenditure, Socioeconomic disadvantage, Account-based gambling data

## Abstract

**Background:**

Gambling and its harmful effects on human health and well-being represent a significant public health concern in many countries, with electronic gambling machines (EGMs) recognized as one of the most detrimental forms of gambling. Previous research has established an association between EGM accessibility, expenditure, gambling harm, and the socioeconomic status (SES) of neighborhoods. However, there is limited understanding of the direct impact of SES and EGM accessibility on individual player expenditures. Prior estimations of expenditure often rely on self-reported data or venue-level revenue statistics. This study uses high spatial resolution socioeconomic data together with individual-level account-based location and expenditure (point of sales) data (71,669 players, 745 EGM venues) to explore the association between EGM accessibility and neighborhood SES and to examine whether the EGM expenditure of neighborhood residents is associated with EGM accessibility and neighborhood SES.

**Data and methods:**

Player account data include information on the home location and expenditure of the entire EGM gambling population across every EGM venue located in the Helsinki region, Finland. High-resolution (250 × 250 m) grid-level data on socioeconomic variables were used to obtain the local socioeconomic conditions of the players. EGM accessibility was estimated for every grid cell using a calibrated gravity model derived from the player account data. Statistical analyses included correlation analysis, spatial autocorrelation analysis, and regression models.

**Results:**

First, significantly higher levels of EGM accessibility were found in areas with lower local SES. Second, regression analysis revealed that both higher EGM accessibility and lower local SES were associated with higher annual losses per adult. These results, in combination with visual and spatial autocorrelation analyses, revealed that accessibility to EGM gambling is highly concentrated, especially in lower socioeconomic neighborhoods with higher levels of EGM expenditure.

**Conclusions:**

The results lay the groundwork for future spatial research on gambling harm, expenditure, accessibility, and SES utilizing detailed account data on the interaction between players and venues. The results underscore the importance of spatial restrictions when regulating EGM accessibility, particularly in areas with vulnerable populations, as a crucial measure for public health and harm prevention. The results also enable targeted gambling harm prevention actions at the local level.

## Background

Gambling and its harmful effects on human health and well-being are increasingly recognized as significant public health and policy issues in many countries [1]. While there is no clear evidence on the directionality, there is interrelation between excessive gambling and multiple negative effects on human health and health behaviors, such as mental health difficulties and substance use [2, 3]. Moreover, gambling is also linked to variety of social harms, including financial hardship, unemployment, and relationship problems [4].

Of the various gambling products, electronic gambling machines (EGMs) have been shown to be among the most harmful forms of gambling, mainly due to the structural features of EGMs, such as speed, near misses and high event frequency combined with attractive audiovisual elements [5, 6]. In addition to structural characteristics, a significant concern is the consistent finding across jurisdictions in Europe, North America and Oceania that the accessibility and availability of EGMs are greater in socioeconomically more disadvantaged neighborhoods than in advantaged ones [7–15]. Neighborhood accessibility and the availability of EGMs have been associated with increased gambling participation, higher rates of gambling harm [5, 16–19] and higher rates of seeking help for problematic gambling [20]. Exposure to gambling is linked to higher expenditure rates [8, 14, 21], which in turn predict harm [22].

In gambling research, availability and accessibility are often used interchangeably to refer to different dimensions of physical exposure to gambling products [23]. Availability is typically measured as the number of terrestrial gambling venues (e.g., casinos, arcades) or as the number of EGMs in a given region [21], while geographical accessibility is understood to be the cost of reaching (e.g., distance, travel time, monetary cost) gambling opportunities [5, 23]. Definitions of accessibility, however, vary with accessibility metrics, which sometimes incorporate several measures, such as density, travel cost, attractiveness, and other possible attributes in the models [24, 25]. The focus on density and distance in gambling research aligns with policy relevance, as both can be regulated to prevent harm. This emphasis on policy-relevant factors is consistent with the criteria outlined in accessibility research [26]. Incorporating both density and proximity to spatial analyses of exposure to gambling opportunities is recommended in gambling research [23].

Several studies show that the accessibility of EGMs is higher in socioeconomically disadvantaged neighborhoods when measured by the distance to the nearest venue [8, 12, 27]. For example, a Canadian study revealed a significant negative correlation between the average walking distance to the nearest EGM venue and both average household income (r = -0.378) and the proportion of individuals aged 20 years or older without a high school diploma (r = -0.307) [27]. A Finnish study revealed that for every 1000 euro increase in median income, there was a 0.06 unit decrease in EGM density [11]. Similarly, numerous studies across various jurisdictions have confirmed higher EGM density in socioeconomically disadvantaged neighborhoods [9, 10, 13, 14, 28], which can lead to greater gambling harm for residents.

Local gambling exposure and neighborhood socioeconomic status have been linked to increased average individual expenditures [8, 14, 21]. An Australian study using venue-level revenue data revealed a 0.5% increase in expenditure per adult for every one-point increase in an index of socioeconomic deprivation, with 40% of this effect attributed to EGM density [8]. Likewise, Grumstrup and Nichols [14], utilizing venue-level revenue data in Illinois, United States, reported that a 1 percentage point increase in the poverty rate corresponded to a 1.47% increase in EGM expenditure per capita and a 1.17% increase in EGM density. Using self-reported expenditure data in Canberra, Australia, Marshall et al. [21] found that individuals living within two kilometers of their regularly visited venue had the highest annual expenditure levels.

Research indicates that local area disadvantage and other contextual factors are likely to have an impact on the strength of the relationship between neighborhood disadvantage, accessibility to EGMs, and expenditure on EGMs [29]. For instance, an Australian study found no consistent spatial correlation between gambling expenditure and local level socioeconomic disadvantage, highlighting how contextual factors can influence such relationships [30]. Gambling expenditures in neighborhoods with gambling venues can vary for many reasons: there can be a small group of players in the neighborhood with elevated levels of spending, or the players visiting the neighborhood may spend on gambling [29, 30]. Whether the higher levels of EGM spending stem from local residents or from people visiting the venues has been examined in studies on the catchments of the venues [18, 21, 29]. Marshall et al. [21] reported that there was considerable variation in the sizes of the catchment areas of different EGM venues. Young et al. [18] reported that one-third of customers visited the closest gambling venue, but individuals with an increased risk of gambling harm were more likely to visit the venues closer to their homes. Furthermore, there is evidence that different venues attract different parts of the population [25].

In this study, we contribute to the existing research in the field by analyzing the associations between local SES, geographic accessibility to EGMs, and actual expenditure on EGMs. Prior research indicates that relatively little is still known about whether higher accessibility to EGMs in a neighborhood is associated with a higher level of expenditure for players residing in the neighborhood. From a methodological viewpoint, data on average expenditure are largely derived from self-reports or venue-level revenue statistics. A typical weakness associated with survey data is subjective bias, while venue-level statistics do not reveal the exact residential locations of the players. Overall, previous studies utilizing player account-based expenditure data [31] or grid data on socioeconomic status are scarce [8, 9]. We are unaware of other studies that have combined actual individual-level interaction data between players and EGMs (stakes, losses), the exact residential addresses of players, and high spatial resolution grid data on socioeconomic status. By utilizing these data sources, we seek to significantly advance the current understanding of the associations among the geographic accessibility of EGMs, expenditure, and socioeconomic status at the local area level.

To summarize, the aims of this study are (1) to examine the association between EGM accessibility and the socioeconomic status of different neighborhoods and (2) to examine whether the EGM expenditure of neighborhood residents is associated with EGM accessibility and neighborhood socioeconomic status.

## Data and methods

### Study context and area

Finland provides an excellent opportunity for the spatial analysis envisaged above. Unlike in most jurisdictions, most noncasino EGMs are widely accessible to people in everyday life environments (e.g., restaurants, grocery stores, kiosks, and gas stations). Additionally, there are separate gambling arcades. The EGMs are operated by the state-owned gambling monopoly company Veikkaus. The minimum legal gambling age is 18 years. Since July 2021, all players have had to authenticate themselves when playing EGMs. Subsequently, every EGM player’s residential address is known, and every player’s interaction with the machines is recorded.

The study area was the Helsinki metropolitan region in southern Finland, which consists of the municipalities of Helsinki, Espoo, and Vantaa, with a total population of 1.2 million in 2022 and a land area of approximately 765 square kilometers. Data on the small municipality of Kauniainen within the borders of Espoo were not available. The study area represents a suitable context for the current study based on the following features: The area contains a large number of EGM venues, it is the largest uniform urban area in Finland in terms of area and population, and it has been experiencing increasing segregation in the last few decades [32, 33], which allows us to capture socioeconomic differences in expenditure and accessibility more distinctly.

### Data sources and filtering

The data for the study consisted of two main datasets: grid data on socioeconomic status (SES) of the adult population at a 250 × 250 m spatial resolution and player account-based gambling data. The player account-based gambling data were obtained from Veikkaus, the national gambling monopoly in Finland. Section 55 of the national Lotteries Act permits the use of gambling register data for research purposes upon request. This right to obtain information also applies to essential personal data. Furthermore, the [anonymized for peer review] has also the right to combine personal data with other data, where this is necessary for processing. The gambling register data were anonymized before being handed over to the researchers. The gambling register data are not publicly available, whereas the population data by map grids are available from Statistics Finland by purchase.

**Fig. 1:**
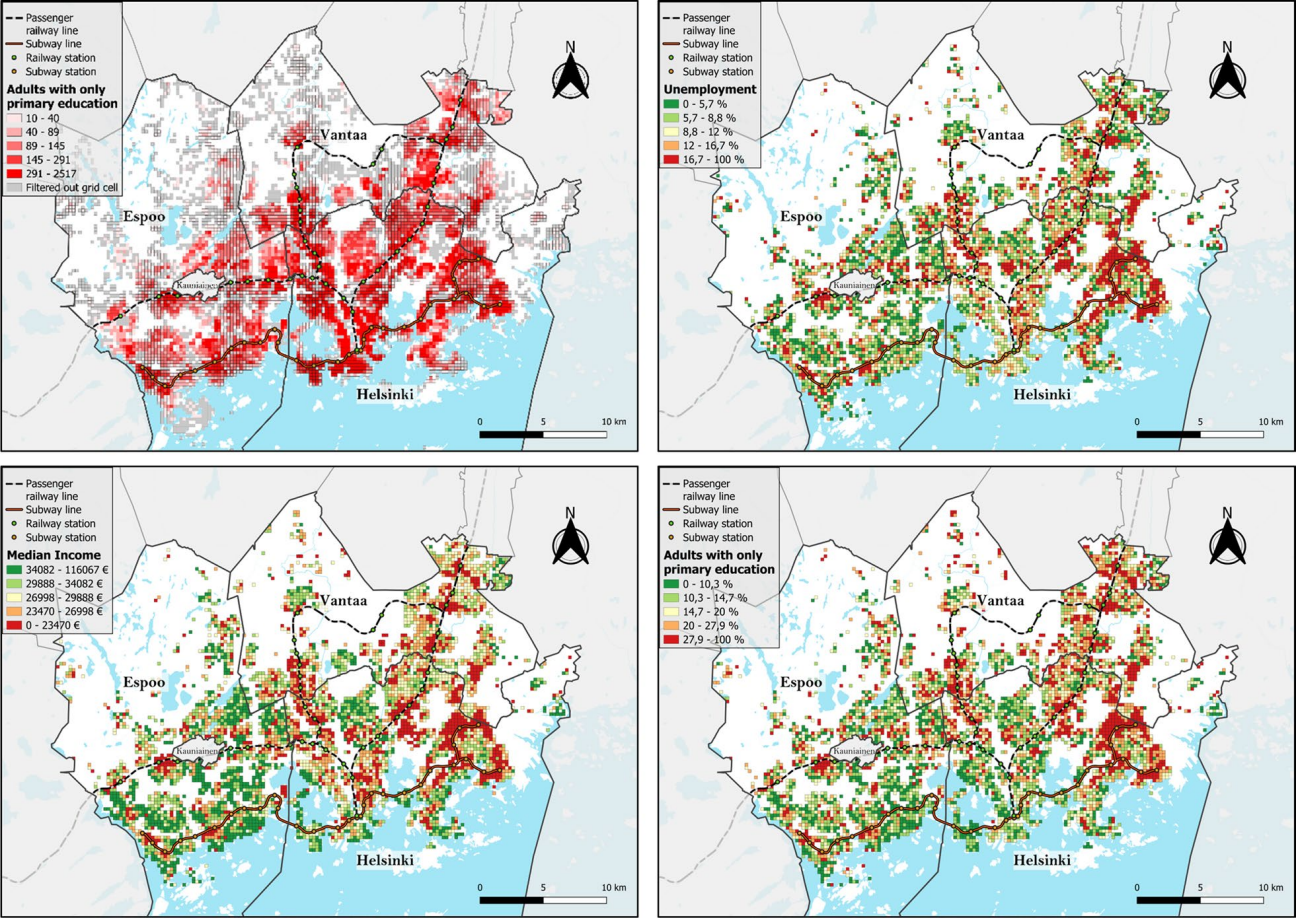
Top-left: Adult population distribution in quintiles with filtered grid cells marked as gray. Top-right: unemployment % in quintiles Bottom-left: Median income in quintiles. Bottom-right: Percentage of adults with only primary level education in quintiles

The total adult population, mean age, number of employed and unemployed individuals, number of people with primary education only, and median net income in the *socioeconomic grid data* were used for further analysis to represent the SES of each grid cell (Fig. 1). Educational level, income and unemployment rate are commonly used in gambling research as indicators of vulnerability to gambling harm [11, 12]; these indicators are available from Statistics Finland and have been widely used in similar studies [9, 13]. Data on the total adult population, mean age and education level were collected for 2021, while employment and income data were collected for 2020. The original dataset included 6,643 cells that had at least one adult inhabitant and a total adult population of 950,309. Due to privacy concerns, socioeconomic data are reported only for cells with an adult population (over 18 years old) of more than 10 people. After including only the grid cells containing all available socioeconomic data and for which walking routes between the grid cell centroids and venues could be calculated along the OpenStreetMap walking network [34], we were left with 4,953 grid cells and a population of 940,397 for further analyses.

**Fig. 2:**
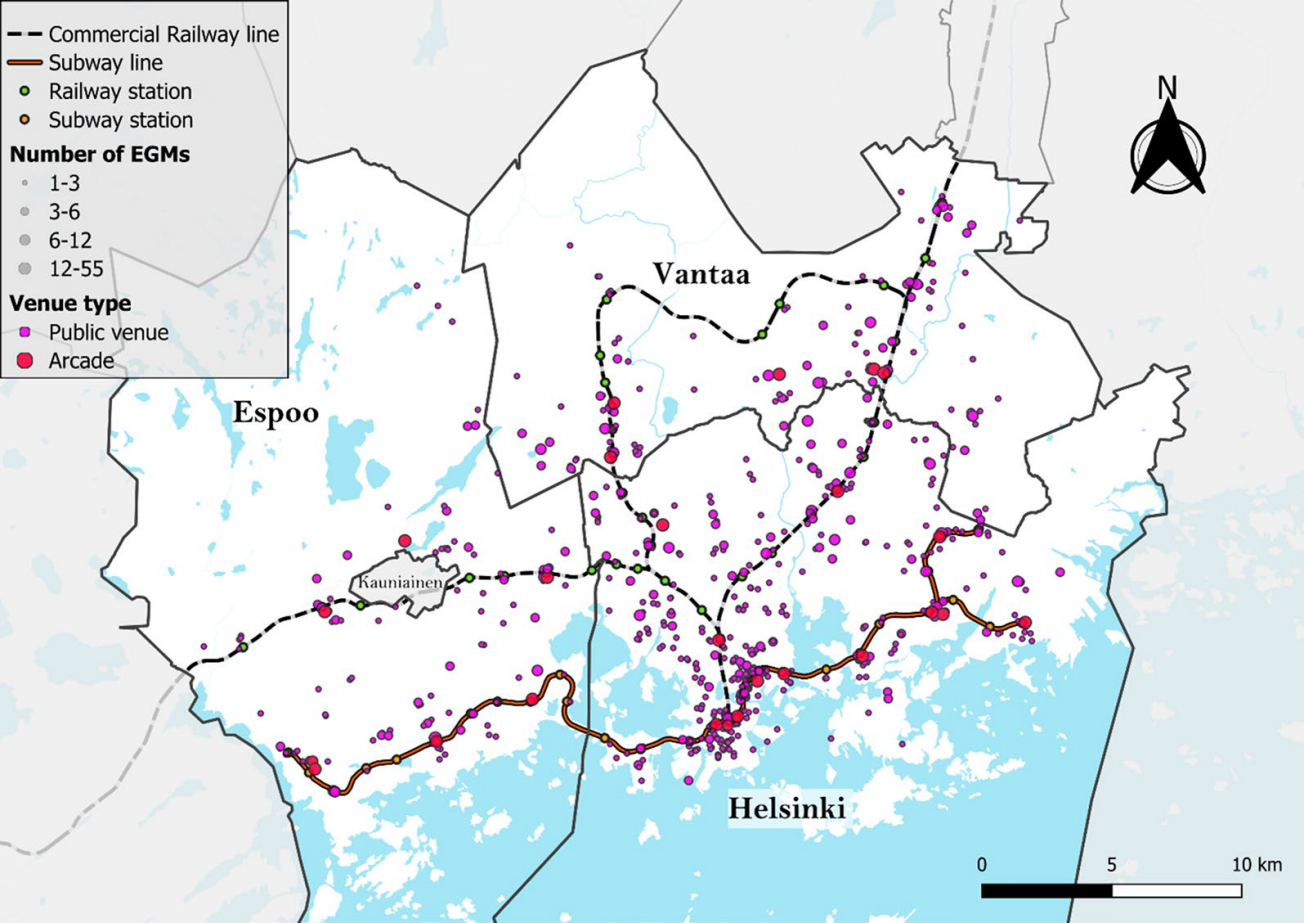
Distribution of EGM venues in the study area. The size of the dot is proportional to the number of EGMs in the venue, and the coloring indicates the venue type

The *gambling data* contained information on the stakes and losses of each individual player residing in the study area to EGM venues located within the study area during the year 2022. Each player and EGM venue also had street-address information attached, which was geocoded to coordinates using a combination of the Nominatim geocoding service [35] and the geocoding service of the National Land Survey of Finland [36]. The data on the number of EGMs in each venue were weighted by their time of operation during the study year, which means that the number of EGMs is expressed as a floating-point number in this study. The original dataset included 72,980 players, 751 venues (including 26 arcades), and a total of 2,554.2 EGMs. After geocoding and filtering out players who did not fall inside the grid cells with socioeconomic information and walking routes, 71,669 players, 745 EGM venues, and 2,397.1 EGMs were included in the final analyses (Fig. 2). Data on players’ stakes, net losses and numbers of players were aggregated and merged with the socioeconomic grid cells by conducting a spatial join between the player-address points and socioeconomic grid cell polygons and summing them together.

## Methods

Data processing and aspatial statistical analyses were performed using the Python programming language and various data analysis packages. Specifically, the *Pandana* Python package version 0.7 [37] was used for calculating the walking network distances between the population grid cells and EGM venues, *statsmodels* version 0.14.0 [38] was used for the regression analyses, and *Lmfit* version 1.2.2 [39] was used to fit different function models against our data points when calibrating the gravity model. Map visualizations were created using QGIS version 3.28.11 [40], and spatial autocorrelation analyses were carried out using GeoDa version 1.22.0.2 statistical software [41].

### Gravity model of accessibility

In this study, both density and distance to EGMs were understood as dimensions of geographic accessibility. Accessibility to EGMs was defined using a gravity model, a widely used method for quantifying interaction or accessibility between locations in geographical research [18, 25]. In the model, EGM accessibility was defined as the number of gambling venues within a prespecified walking distance from the grid cell centroid, in which the impact of each venue on accessibility was weighted by a function of distance decay and the number of EGMs in each venue. Possible additional dimensions of accessibility, such as personal constraints or preferences and competition effects, were not considered in the model.

No all-purpose functions or parameters for calculating accessibility using the gravity model have been established, and determining the function form, strength of distance decay, and other weighting parameters are commonly assigned based on the research context and available data. In our gravity model, actual interaction data between people and EGMs at different distance thresholds were used to determine venue catchment areas and the distance decay effect within these areas. The distance decay effect on accessibility was determined by examining the average adult expenditure per available EGM venue in each grid cell with available venues in 100-m distance bins. Expenditure to EGMs was chosen as the distance decay proxy instead of visitation rates or other possible metrics, as this was considered to capture the intensity of gambling more profoundly.

**Fig. 3:**
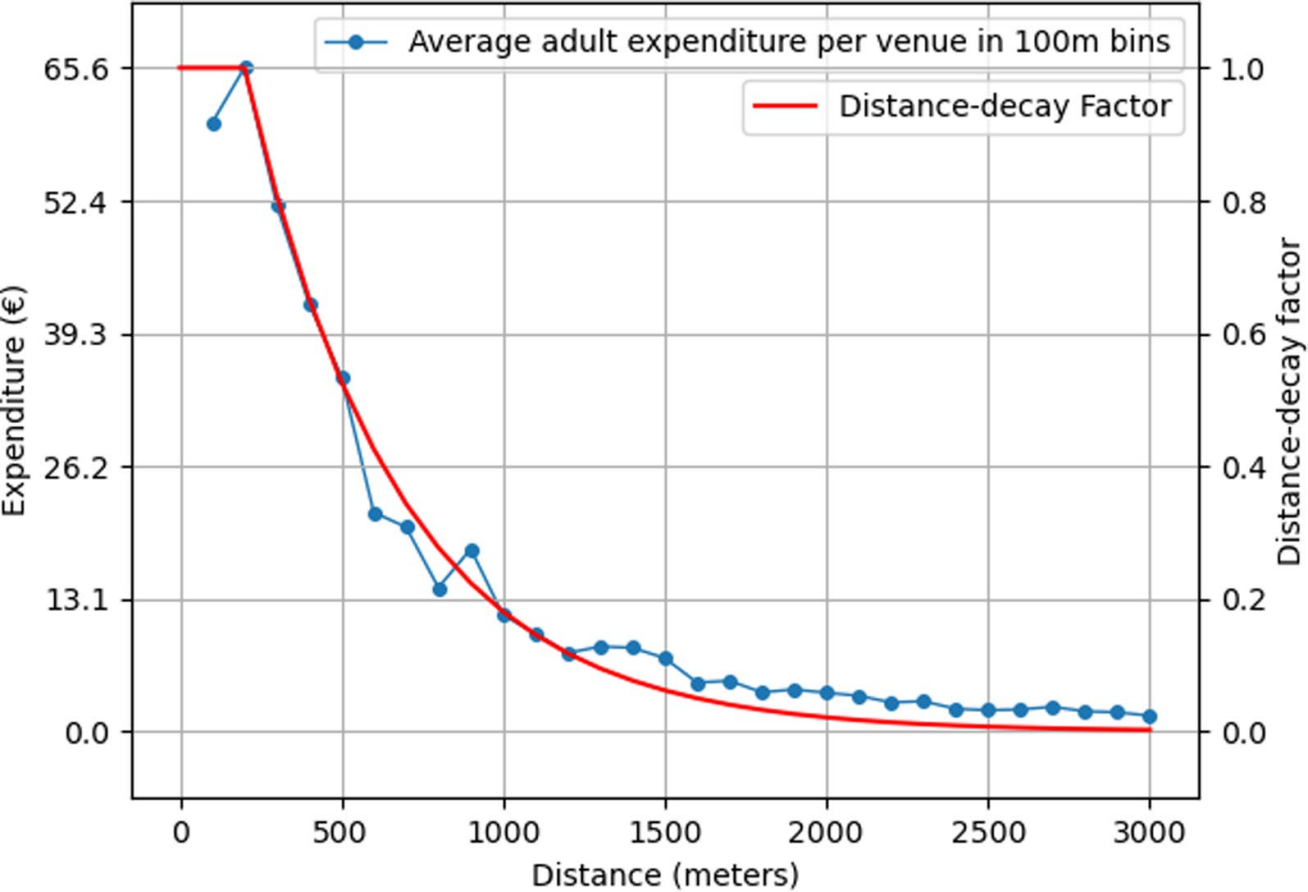
Fitted distance decay curve against the average adult expenditure per available venue in each grid cell with available venues in 100 m distance bins

Based on the expenditure in different distance bins, 3,000 m was selected to represent the venue catchment area because the average expenditure per venue in bins beyond this distance threshold was negligible. Distance decay within the catchment area was calculated by fitting different parameterized models against the expenditure data using least squares minimization, of which an exponential model was determined to be the best fitting model for our data based on the acquired R^2^ value of 0.979 (Fig. 3). The effect of distance decay was calculated only for venues farther away than 200 m, as this was the distance at which the average expenditure peaked, and venues located within this threshold were assigned a distance decay factor of 1 (no decay).

To consider the effect of the number of EGMs in a venue on accessibility, a logarithmic regression curve was fitted between expenditure within the venue catchment area and the number of EGMs in the venue. This procedure was chosen because simply using the number of EGMs in a venue has been shown to overestimate their effect on accessibility, and having multiple venues with a small number of EGMs exposes people more to gambling compared with a few venues with a high number of EGMs [25].

Using the aforementioned methods, the number of accessible EGMs for each grid cell was defined with the following function:

$${A}_{i}=\sum_{j}f({y}_{j}, {d}_{ij})$$, where. $$f({y}_{j}, {d}_{ij})={log}_{\beta }({1+y}_{j})$$, when d_ij_ <  = 200 m.

$f({y}_{j}, {d}_{ij})={{log}_{\beta }({1+y}_{j})*e}^{(-\frac{{d}_{ij}-200}{\tau })}$, when 200 m < d_ij_ <  = 3000 m. where A_i_ is the EGM accessibility for each grid cell i, y_*j*_ is the number of EGMs in each venue j, d_ij_ is the network distance between grid cell i and venue j in meters, τ is the constant distance decay factor of 467.79, and β is the constant logarithm base indicating the effect of each additional EGM in a venue on accessibility (1.76).

### Indices of socioeconomic status and EGM vulnerability

The SES of each grid cell was defined with an SES index constructed of three indicators: the *percentage of unemployed individuals*, the *percentage of people with only primary education,* and the *yearly median net income.* The variables were first normalized to modified Z-scores to bring them to a common scale, after which the sign of the Z-score for the median income was inverted and the variables were summed together to create the index. The index values vary between -14.90 and 21.78, with higher values indicating a lower SES. On average, an increase of one in the index value equaled a 2.2% increase in unemployment, a 3.2% increase in the share of the basic educated population, and a 2,025€ decrease in median net income.

To facilitate the spatial analysis of the overlap between SES and EGM accessibility, an *EGM vulnerability index* was created following the methodology previously used by Rintoul et al. [5]. The vulnerability index was calculated by dividing the aforementioned SES index and EGM accessibility values into deciles according to the number of grid cells and multiplying the decile numbers to obtain index values for each grid cell ranging from 1 (least vulnerable) to 100 (most vulnerable).

### Analysis

Descriptive statistics were used to examine the association between SES and accessibility. Spatial patterns of accessibility as measured by the gravity model, SES, and EGM vulnerability index were examined visually using choropleth maps and statistically by conducting a spatial autocorrelation analysis using global and local Moran’s I methods. Descriptive statistics and graphs were used to examine variations in expenditure by distance, SES, and accessibility, and a linear ordinary least squares regression analysis (OLS) was conducted to examine the association between the SES index and EGM expenditure per adult. OLS was chosen because it is a flexible, easily interpreted, widely used, and suitable method for analyzing the association between expenditure, accessibility, and SES [11]. Accessibility to EGMs, adult population, and mean age were included in the regression model as independent variables.

## Results

### Spatial association between accessibility and socioeconomic disadvantage.

The descriptive results of the EGM venue, player and SES data are described in Tables 1 and 2. An analysis of the geographical distribution of venues and EGMs shows that the number of reachable venues and EGMs is highest in the most disadvantaged neighborhoods (grid cells), especially within smaller distance thresholds (Table 3). For example, there were on average 1.6 venues and 7 EGMs within a network distance of 500 m from grid cells belonging to the most disadvantaged quintile (quintile 5), while there were only 0.4 venues and 1.2 EGMs within the same distance from the most advantaged quintile (quintile 1). As the distance increases, the difference in accessible venues and EGMs between the most disadvantaged and the most advantaged neighborhoods decreases. However, even within a 2,000-m distance, the difference was clear between the most disadvantaged quintile of the neighborhoods and the most advantaged quintile.

**Table 1:** Descriptive statistics of EGM venues (n = 745) and players (n = 71,669) in the study area

	Venues (n = 745)			Players (n = 71,669)	
	Stakes (EUR)	Losses (EUR)	EGMs	Stakes (EUR)	Losses (EUR)
*Mean*	430,173	37,636	3.2	4,472	391
*Std*	772,018	65,362	4.9	15,105	1,267
*Min*	315	−11.2	1	0.2	−7,218
*Median*	195,690	16,752	2	187	23.2
*Max*	9,401,826	750,243	54.1	303,045	17,803
*Sum*	320,478,577	28,039,048	2,397	320,478,577	28,039,048

**Table 2:** Descriptive statistics of population grid cells (n = 4953)

	*Adult Population*	*Unemployment (%)*	*Median Annual Net Income (EUR)*	*Basic Education %*	*SES Index*	*Mean Age*
*Mean*	189.9	11.5	29,144	19.5	0.3	40.3
*Std*	227	7.8	7,028	11.1	2.9	6.4
*Min*	10	0	0	0	−14.9	19
*Median*	113	10.3	28,455	17.2	−0.2	40
*Max*	2,517	100	116,067	100	21.8	82

**Table 3:** Descriptive statistics and the cumulative average number of available venues and EGMs within different distance thresholds and SES population quintiles

SES Quintile	Cells	Population	Registered Players	SES-index Range	Unemployment (%)	Basic Educated (%)	Mdn. Income (EUR)	250 m		500 m		1000 m		2000 m		3000 m	
								Venues	EGMs	Venues	EGMs	Venues	EGMs	Venues	EGMs	Venues	EGMs
1	1,690	188,127	8,196	−14.9 to −1.1	6.8	10.6	34,433	0.1	0.3	0.4	1.2	2.3	6.4	10.4	31.9	22.8	72.1
2	1,018	188,830	11,216	−1.1–0.2	9.6	13.9	28,599	0.2	0.4	0.8	2	3.7	10.1	14.3	44.5	29.9	94.3
3	854	187,510	14,391	0.2–1.7	12.4	19	25,697	0.3	0.6	1.2	3.3	4.3	13.2	15.9	53.2	31.9	106.8
4	710	187,904	17,581	1.7–3.6	15.6	25.5	23,085	0.4	1.1	1.4	4.8	4.8	17.2	15.6	56.2	30	105.9
5	681	188,026	20,285	3.6–21.8	23.1	37.7	20,536	0.4	1.6	1.6	7.1	5.3	22.8	14.9	57.7	28.2	106.9

**Fig. 4:**
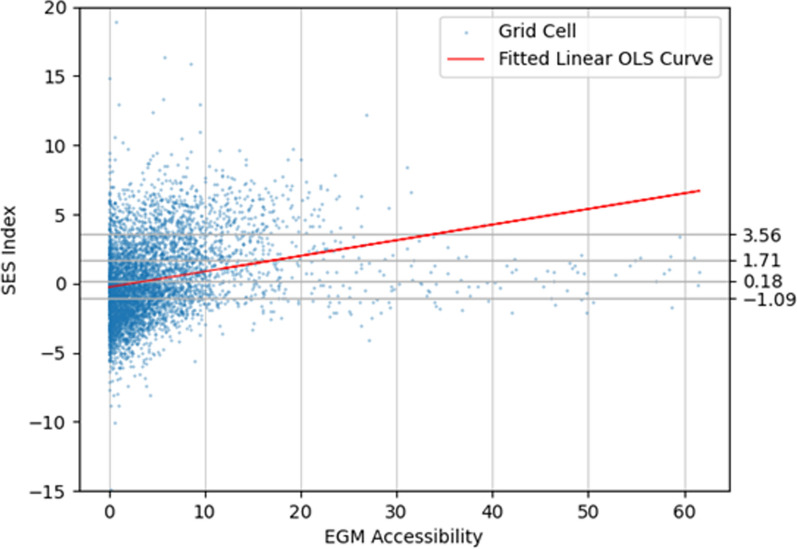
Scatter plot and a fitted linear OLS curve between EGM accessibility and the SES index (Pearson correlation coefficient = 0.259, p < 0.001). The SES quintile boundaries are marked on the x-axis

There was a significant linear positive correlation (r_p_ = 0.259, p < 0.001) between accessibility measured by the gravity model and the neighborhood SES index. Thus, the higher the accessibility is, the more disadvantaged the neighborhoods are. This is discernible in Fig. 4, where accessibility is presented against the SES of the grid cells using a scatterplot and a fitted linear ordinary least squares (OLS) curve.

**Fig. 5:**
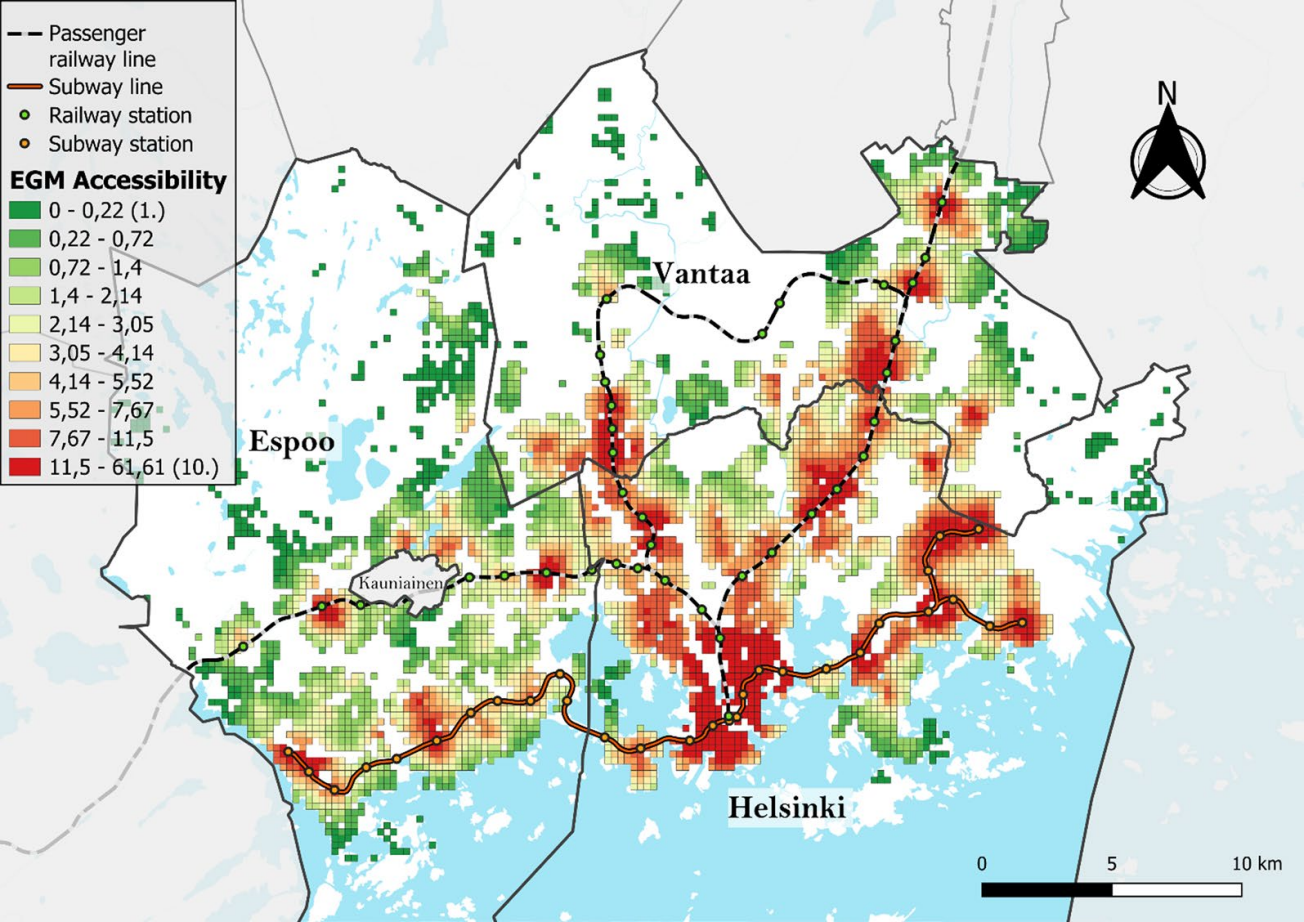
EGM accessibility deciles (equal number of grid cells in each class) used to calculate the vulnerability index

**Fig. 6:**
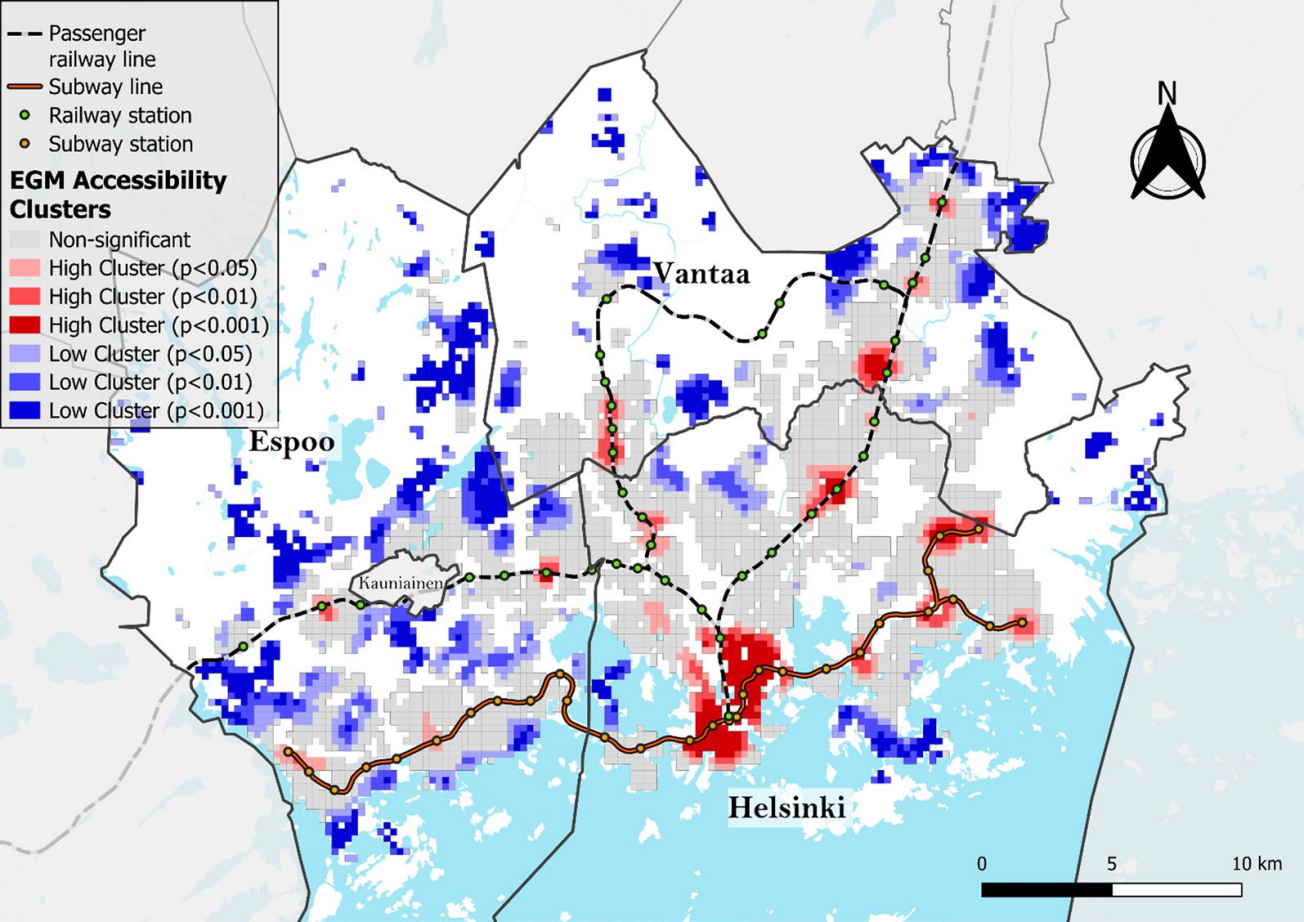
Moran's I clusters of EGM accessibility using a 1st-order queen contiguity spatial weight matrix and different pseudo p-value thresholds (Global Moran’s I = 0.940, z-value = 105.05, p < 0.001)

When examining the spatial patterns of EGM accessibility using choropleth and Moran’s I cluster maps, several distinct high EGM accessibility clusters were identified (Figs. 5 and 6) with notable global spatial autocorrelation (Moran’s I = 0.940). The densely populated central business district of Helsinki in the south was identified as the largest accessibility cluster. Other areas with high accessibility were areas near the EGM arcades located alongside the subway or railway lines north and eastward from the central business district. Specifically, major high-accessibility clusters emerge in several neighborhoods in Helsinki (Malmi, Kontula and Itäkeskus) and in Vantaa (Myyrmäki and Tikkurila), all of which are neighborhoods containing a subway or a railway station as well as an arcade and several smaller venues. Apart from some small clusters of high accessibility centering around a few arcades, Espoo was characterized by low EGM accessibility, with a median accessibility value of 1.57 compared to 2.48 for Vantaa and 5.55 for Helsinki.

**Fig. 7:**
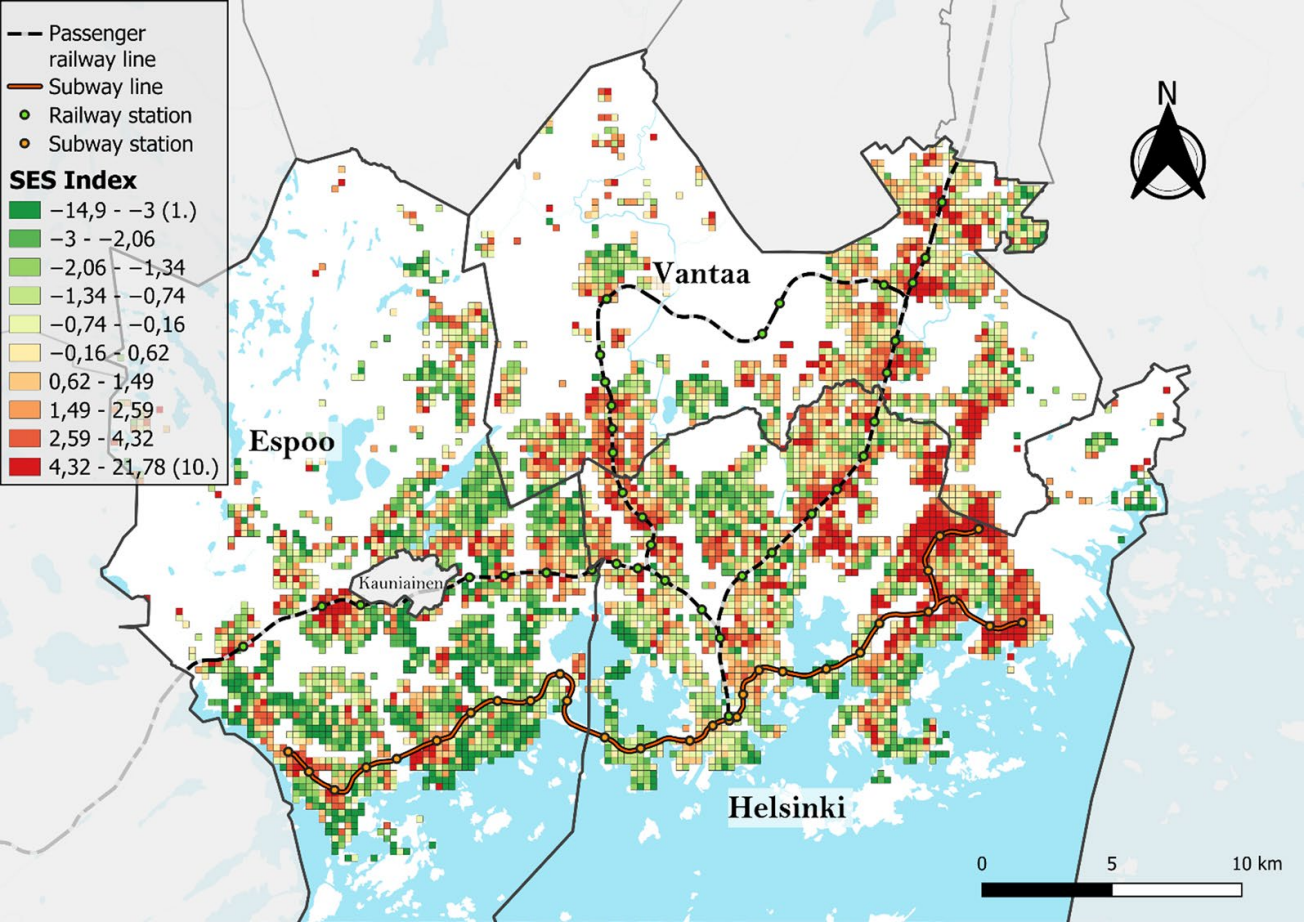
SES index deciles (equal number of grid cells in each class) used to calculate the EGM vulnerability index

**Fig. 8:**
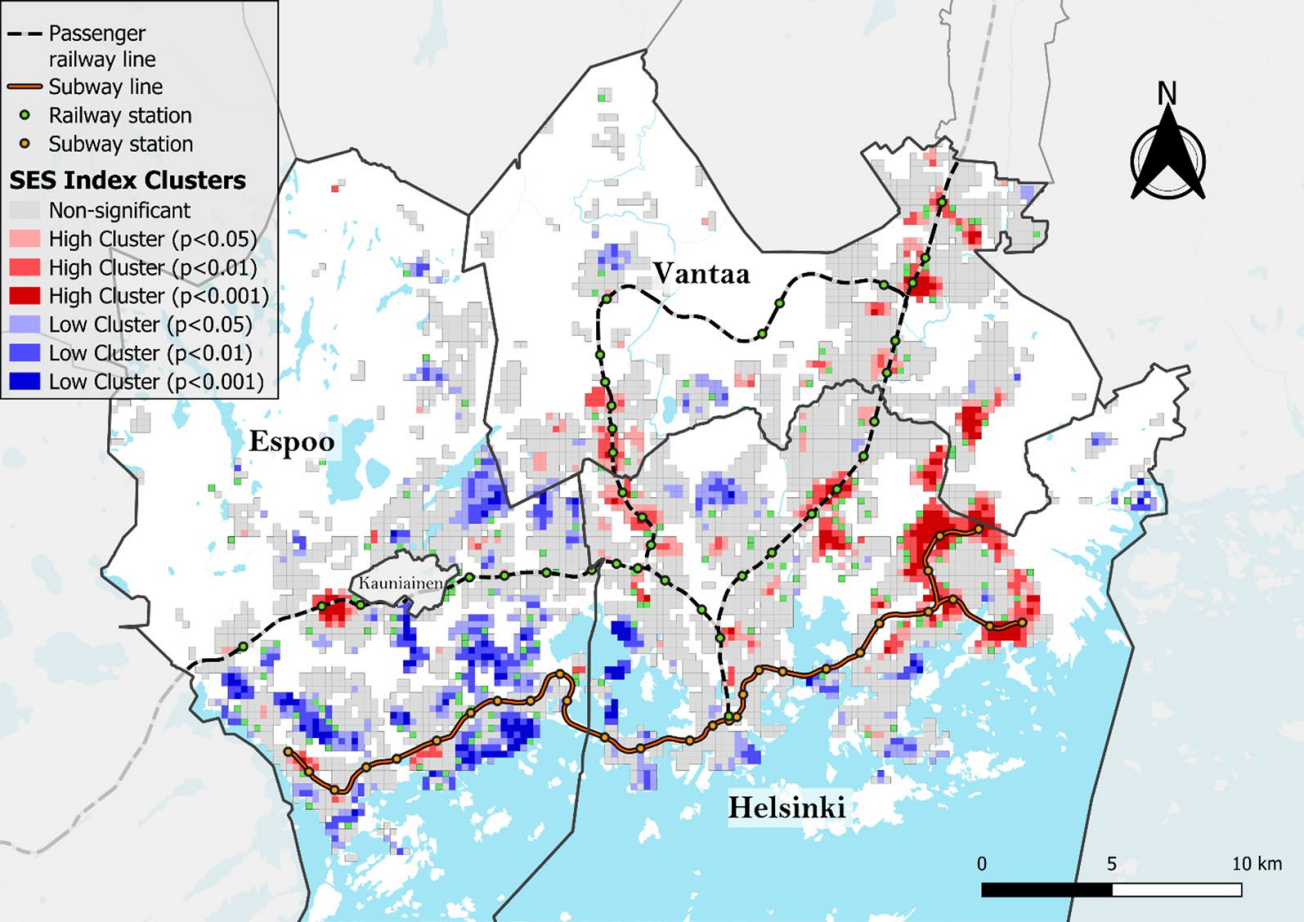
Moran's I clusters of SES index values using a 1st-order queen contiguity spatial weights matrix and different pseudo p-value thresholds (Global Moran’s I = 0.437, z-value = 48.89, p < 0.001)

**Fig. 9:**
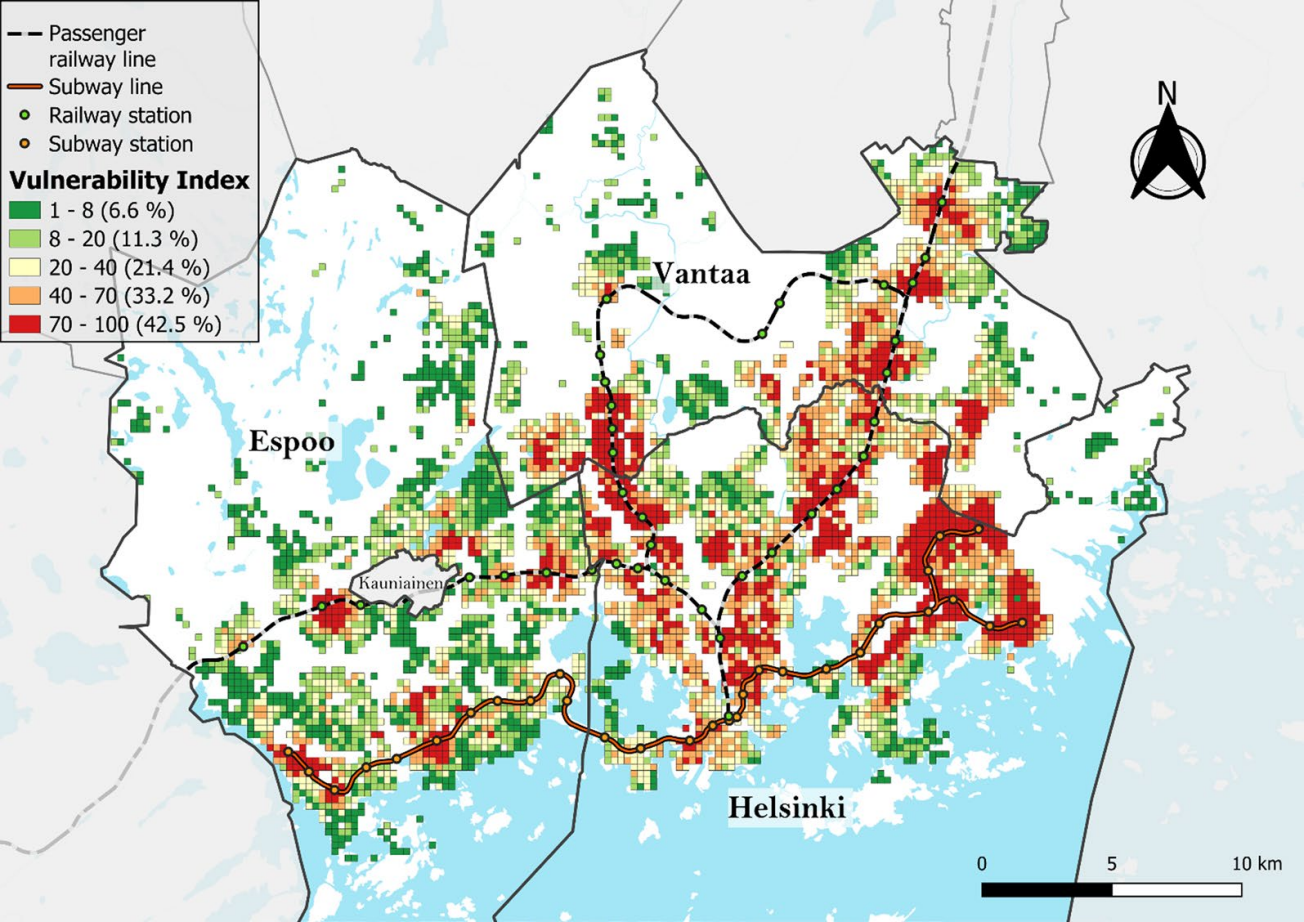
EGM vulnerability index quintiles (equal number of grid cells in each class) with population distribution depicted in brackets

**Fig. 10:**
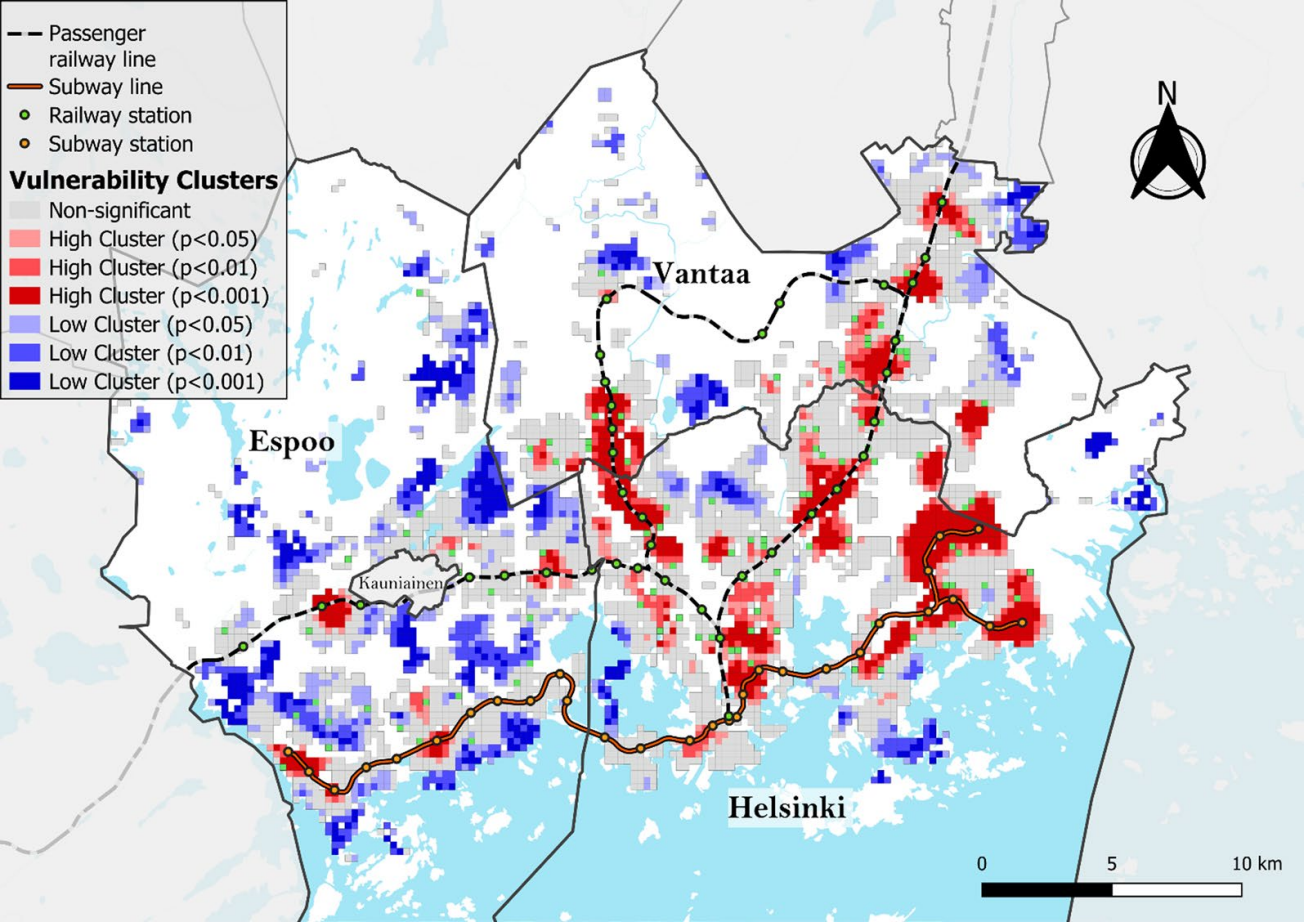
Moran's I clusters of the EGM vulnerability index using a 1st-order queen contiguity spatial weights matrix and different pseudo p-value thresholds (Global Moran’s I = 0.759, z-value = 87.9, p < 0.001)

The mapping and cluster analyses of the SES index values revealed that disadvantaged neighborhoods are located mainly in Helsinki and Vantaa and have similar cluster patterns to the EGM accessibility, with the Helsinki central business district being a notable exception, with no high or low clusters identified (Figs. 7 and 8). The mapping of the EGM vulnerability index and its local spatial autocorrelation confirms this observation (Figs. 9 and 10). Helsinki and Vantaa have large areas with some overlap between high EGM accessibility and low socioeconomic status, making their population particularly vulnerable to EGM gambling, while Espoo is characterized by low or nonsignificant vulnerability aside from areas near arcades located along railway or subway lines (Figs. 9 and 10). Around the eastern ends of the subway line are especially large areas of high vulnerability, with extensive areas identified as local clusters. The global Moran’s I value of 0.759, along with the uneven population distribution across the vulnerability quintiles (42% of the total population belonging to the highest vulnerability quintile and 6.5% in the lowest), indicates that EGM vulnerability in the study area is highly clustered both in terms of area and population.

#### Spatial patterns and relationship between EGM expenditure and neighborhood disadvantage

The analyses show that EGM gambling losses were concentrated in neighborhoods with the highest levels of socioeconomic disadvantage. The absolute annual losses per adult were on average 57 euros in the SES quintile with the highest disadvantage compared to the 11 euros in the most advantaged quintile (Table 4). Of the total expenditure, 39 percent was from people living in the most disadvantaged neighborhoods, while 7 percent stemmed from the most advantaged neighborhoods (Table 4). Moreover, the average annual losses per player followed a similar pattern: 532 euros in the most disadvantaged neighborhoods and 247 euros in the most advantaged neighborhoods. Setting these figures in proportion to the median income in the different neighborhoods shows that the average expenditure of players was 2.6 percent of the median income in the most disadvantaged neighborhoods and 0.7 percent in the most advantaged neighborhoods.

**Table 4:** Cumulative adult annual losses, share of SES quintile losses on EGMs, and share of total losses on EGMs within different distance thresholds and SES population quintiles

*SES Quintile*	***250 m***			***500 m***			***1000 m***			***2000 m***			***3000 m***			***Total***		
	*Losses per Adult (EUR)*	*% of SES-Quintile Losses Losses*	*% of Total Losses*	*Losses per Adult (EUR)*	*% of SES-Quintile Losses*	*% of Total Losses*	*Losses per Adult (EUR)*	*% of SES-Quintile Losses*	*% of Total Losses*	*Losses per Adult (EUR)*	*% of SES-Quintile Losses*	*% of Total Losses*	*Losses per Adult (EUR)*	*% of SES-Quintile Losses*	*% of Total Losses*	*Losses per Adult (EUR)*	*% of SES-Quintile Losses*	*% of Total Losses*
*1*	0.3	3.1	0.2	1	9.6	0.7	2.4	22.8	1.6	4.3	40.5	2.9	5.8	54.2	3.9	10.8	100	7.2
*2*	1	5.4	0.6	2.5	14.1	1.7	5.3	29.5	3.5	8.5	47.4	5.7	10.2	57.1	6.8	17.5	100	11.8
*3*	1.3	5.4	0.9	4.7	19.1	3.1	8.3	33.8	5.5	12.5	50.8	8.3	14.9	60.5	9.9	25	100	16.8
*4*	3.8	9.8	2.5	8.8	22.8	5.9	15.9	41.2	10.7	22.4	58	15	25.2	65.3	16.9	38.4	100	25.7
*5*	5.4	9.3	3.6	14.3	25	9.6	24.8	43.1	16.6	32.5	56.6	21.8	36.3	63.2	24.3	57.4	100	38.5

**Fig. 11:**
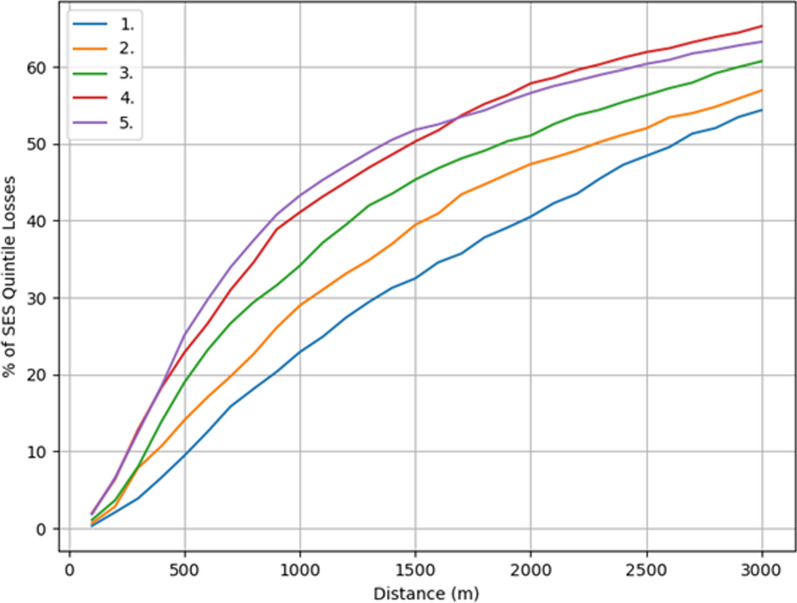
Cumulative percentage of annual money lost to EGMs as distance increases between player and venue locations in the different SES quintiles

Overall, approximately half of the losses to EGMs occurred within a network distance of 2,000 m from the players’ home grid cells. However, there are discernible differences in the spatial distribution of losses from a socioeconomic point of view (Table 4 and Fig. 11). The distribution of losses was highly skewed toward closer distances in the two lower SES quintiles, while the losses were spread more evenly across different distances in the higher ones. For example, only 9.6% of the losses in the most advantaged SES quintile of neighborhoods occurred within 500 m from home, while for the most disadvantaged SES quintile, the same percentage was 25.0%. The spatial differences are even more stark when examining absolute annual losses per adult; for example, within the distance threshold of 1,000 m, people living in the most disadvantaged neighborhoods lost on average ten times more money on EGMs than those in the most advantaged neighborhoods. The difference in relative losses between SES quintiles decreased as distance increased toward the 3,000-m mark, but the absolute losses were still heavily swayed toward the lower SES quintiles regardless of the distance threshold.

**Fig. 12:**
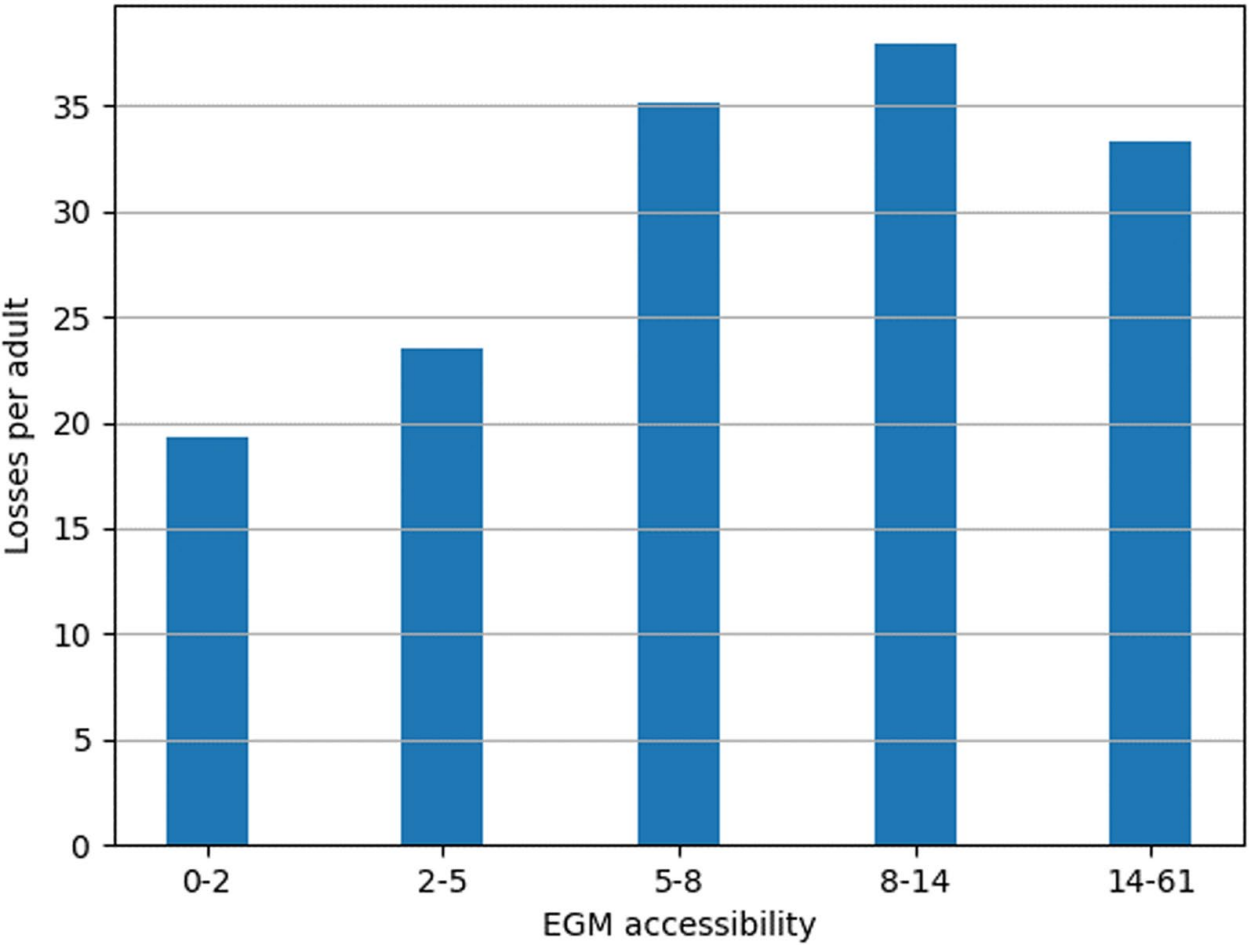
Annual losses per adult in grid cells belonging to different EGM accessibility classes divided by population quintiles

Figure 12 illustrates how accessibility measured by the gravity model and the yearly losses per adult are related. The losses per adult increase steadily as EGM accessibility increases, and the yearly losses per adult were clearly highest in the 7–12 accessibility range. However, the annual losses per adult starkly decrease in the highest accessibility class. A visual analysis revealed that these areas were located mostly in the central business district of Helsinki, with a large number of venues and EGMs but also a relatively high SES compared to other areas with high EGM accessibility.

A regression analysis shows that the SES index is strongly (p < 0.001) associated with the average level of EGM expenditure per adult (Table 5): when the value of the index increases by one, the average annual losses increase by 4.87 euros (Model 1). The same applies to accessibility: the higher the accessibility index is, the higher the level of expenditure (Model 2). Looking at the SES index and accessibility together in Model 3, both coefficients decrease, and there is only a slight increase in the explanatory power of the model (R^2^ = 0.085), indicating some amount of multicollinearity between the two, which was expected based on the accessibility analysis conducted previously. In Model 4, the population and mean age in each grid cell were used as control variables. In this model, the effect of SES remained unchanged, while accessibility increased the losses per adult, which is likely due to multicollinearity between accessibility and population (both have a VIF of 1.6 in the final model). Of the control variables, the population within a grid cell had a negative impact on losses (71 more people equaled a 1€ decrease in net losses per adult), while the mean age had no statistically significant effect. The final model shows that the annual losses per adult increase by 4.80 euros when the SES index increases by one. Overall, the area-level SES is strongly associated (p < 0.001) with the losses of adults on EGMs even when considering accessibility and control variables.

**Table 5:** Ordinary least squares regression of annual EGM losses per adult

Dependent variable: annual losses per adult				
	Model 1	Model 2	Model 3	Model 4
**CONSTANT**	24.4073 (23.119–25.695)	21.2937 (19.639–22.949)	22.5370 (20.930–24.144)	35.1421 (19.616–50.669)
**SES Index**	4.8701 (4.163–5.577) ***		4.6418 (3.916–5.367) ***	4.8016 (4.036–5.567) ***
**EGM Accessibility**		0.9087 (0.704–1.113) ***	0.3841 (0.217–0.551) ***	0.6876 (0.453–0.922) ***
**Population**				−0.0146 (−0.022 to −0.007) ***
**Mean Age**				−0.2832 (−0.654–0.087)
**R**^**2**^	0.082	0.015	0.084	0.088
**Adj. R**^**2**^	0.082	0.015	0.084	0.087

***p ≤ 0.001; 95% confidence intervals are in brackets

## Discussion

By utilizing player account-based gambling data on expenditures, locations of both expenditures and residences, and high spatial resolution socioeconomic grid data, this study aimed to examine the spatial associations between EGM accessibility and neighborhood SES and whether EGM expenditure, EGM accessibility, and local-level SES are associated. In regard to the association between EGM accessibility and neighborhood SES, the results demonstrated a clear positive correlation between EGM accessibility and neighborhood disadvantage. The number of accessible EGM venues and machines was highest in disadvantaged neighborhoods. Furthermore, vulnerable areas characterized by both high accessibility to EGMs and high levels of disadvantage were observed to form several clusters around arcades in the study area. When analyzing the relationships between EGM accessibility, neighborhood SES, and expenditure, the findings revealed that the highest overall expenditure per adult on EGMs occurred in the most disadvantaged neighborhoods, with nearly 40% of total losses originating from residents in the most disadvantaged quintile of the neighborhoods. Expenditure within the most disadvantaged neighborhoods exhibited greater spatial concentration compared to more advantaged neighborhoods, with EGM gambling occurring much closer to home in these areas. Finally, regression analysis showed that higher accessibility of EGMs and socioeconomic disadvantage are both associated with higher average annual losses per adult at the local level.

The finding of high exposure to EGMs in the most disadvantaged neighborhoods is in line with previous studies in other areas [5] as well as with a previous Finnish study [11]. Large parts of the population living in disadvantaged neighborhoods are thus exposed to EGMs. In addition, due to their disadvantaged SES, the identified clusters of high EGM vulnerability located in the suburbs of the Helsinki region are also likely to be areas with high availability of other products with health risks, such as tobacco [42]. Therefore, exposure to EGMs can cause an added health burden in a living environment in which multiple health- and welfare-related behaviors cooccur. Serious public health interventions need to consider the burden caused by the concentration of EGM gambling opportunities.

The people in the most disadvantaged neighborhoods live closer and lose more money to EGMs, especially to machines close to their homes, than do those in more advantaged neighborhoods. A similar finding was reported in an Australian study in which expenditure was highest among players living within 2,000 m of a regularly visited EGM venue [21]. Our findings further confirm that expenditures on gambling are regressive [14] and that the higher accessibility truly translates into greater expenditures on local EGMs. Thus, it is likely that the disproportional accumulation of high EGM accessibility and high EGM expenditure in the same neighborhoods can contribute to income inequality and therefore increased neighborhood segregation.

The primary reason for the high accessibility of EGMs in Finland is the lax regulation of the placement of EGMs with the monopoly operator given the freedom to decide where to place the machines. Only the maximum numbers of EGMs and arcades in the whole country are regulated, leaving much discretionary power to the state-run gambling monopoly (Veikkaus) on the placement of arcades and EGMs. The result of this liberal approach is, as the results of this study show, that there are very vulnerable neighborhoods with high accessibility to EGMs and higher EGM expenditures.

The absolute number of noncasino EGMs decreased significantly (from 21,500 to 12,000) in Finland between 2019 and 2022. Largely due to the introduction of mandatory player identification and the COVID-19 pandemic, the overall losses also decreased from 680 million euros to 165 million euros during the same period. The likely consequence is that the potential harm of EGMs to the health and well-being of the population living in the study area has decreased.

### Study strengths and limitations

Unlike previous research in the field, the motivation behind this study, which also stands as its major strength, lies in the utilization of extensive and reliable gambling register data. Consequently, the results remained unaffected by typical weaknesses associated with survey data, such as subjective bias and laboriousness. Furthermore, the gambling expenditure data encompassed the entire EGM gambling population within the study area. By utilizing account-based data on the actual gambling behavior of every EGM player, we were able to examine expenditures and losses at different distances between homes and venues and refine our gravity model without the need to rely on laborious data collection procedures, such as population surveys with self-reported information on expenditures. Access to accurate interaction data is rare in gambling research, and many previous studies that have attempted to define EGM catchment areas using a gravity model approach have used either hypothetical function parameters and variables [27, 29] or samples of interactions, such as survey data on visitation rates [25]. By using this interaction data, considered as big data, we contribute to the current research trend in geographical studies that utilizes novel spatial big data sources for delineating accessibility models [43, 44]. Similarly, another strength was the use of reliable and impartial high spatial resolution socioeconomic data provided by Statistics Finland.

However, this study is not without limitations. First, the study covered only urban areas, and it is unclear how applicable the results are for sparsely populated rural areas [45]. Moreover, the generalizability of the findings to other jurisdictions should be made with caution. Similarly, the causal mechanisms generating the associations found here remain unknown. The inclusion and consideration of travel times and monetary costs for different modes of transport could also have improved the accessibility analyses [46, 47]. This would require that the ease-of-use and local capabilities for utilizing different transport modes be considered, for example, rates of car ownership and financial limitations on travel, which we considered to be beyond the scope of this research. Finally, we did not have exact information on the openings and closings of arcades and venues as there were still some COVID-19 restrictions in effect in the winter of 2022 which might have an effect on the observed expenditure and if available, could have been used to weight the accessibility of each venue accordingly.

## Conclusions

Our results show that both exposure to and expenditures on EGM gambling is higher in neighborhoods with high socioeconomic disadvantage. Without regulation, such concentrations of EGM gambling opportunities are difficult to avoid. Therefore, from the point of view of public health and gambling harm prevention, it is necessary to address the spatial concentration of gambling opportunities in vulnerable neighborhoods. The spatial concentration of EGMs can be mitigated through spatial regulation. For instance, it is feasible to establish regulations setting a maximum limit on the number of EGMs within a defined geographic area and imposing minimum distances between EGM venues or other locations frequented by vulnerable population groups [10, 30]. It is a public health priority for society to protect people living in the most vulnerable areas and thus promote equality in an economically and socially sustainable way. The results and methods employed in this study may help in targeting preventive actions for gambling harm at a more localized level and in monitoring the impact of these actions.

## Data Availability

The population data by map grids are available from Statistics Finland by purchase. The individual level player account data supporting this study are not publicly available based on legislation defined in the Sect. 55 of the Lotteries Act.

## Abbreviations

EGM: Electronic gambling machine
OLS: Ordinary least squares
SES: Socioeconomic status
